# Surgical experience in repairing the right common carotid artery and the right internal jugular vein after ECMO in neonates: early clinical results

**DOI:** 10.1186/s13052-023-01556-y

**Published:** 2023-11-10

**Authors:** Qi-Liang Zhang, Xiu-Hua Chen, Si-Jia Zhou, Yi-Rong Zheng, Hua Cao, Qiang Chen

**Affiliations:** Department of Cardiac Surgery, Fujian Children’s Hospital (Fujian Branch of Shanghai Children’s Medical Center), College of Clinical Medicine for Obstetrics & Gynecology and Pediatrics, Fujian Medical University, Fuzhou, China

**Keywords:** Neonates, ECMO, Vascular anastomosis, Early results

## Abstract

**Background:**

The purpose of this study was to summarize the early clinical results and surgical experience of repairing the right common carotid artery and the right internal jugular vein after ECMO treatment in neonates.

**Methods:**

We retrospectively collected the clinical data of 16 neonates with circulatory and respiratory failure who were treated with ECMO via the right common carotid artery and the right internal jugular vein in our hospital from June 2021 to December 2022. The effects of repairing the common carotid artery and internal jugular vein were evaluated.

**Results:**

All 16 patients successfully underwent right cervical vascular cannulation, and the ECMO cycle was successfully established. Twelve patients were successfully removed from ECMO. The right common carotid artery and the right internal jugular vein were successfully repaired in these 12 patients. There was unobstructed arterial blood flow in 9 patients, mild stenosis in 1 patient, moderate stenosis in 1 patient and obstruction in 1 patient. There was unobstructed venous blood flow in 10 patients, mild stenosis in 1 patient, and moderate stenosis in 1 patient. No thrombosis was found in the right internal jugular vein. Thrombosis was found in the right common carotid artery of one patient.

**Conclusion:**

Repairing the right common carotid artery and the right internal jugular vein after ECMO treatment in neonates was feasible, and careful surgical anastomosis techniques and standardized postoperative anticoagulation management can ensure early vascular patency. However, long-term vascular patency is still being assessed in follow-up.

## Background

Extracorporeal membrane oxygenation (ECMO) has been used to treat neonatal circulatory and respiratory failure for more than 50 years, since the 1970s. ECMO technology is well-developed and widely used in patients with severe cardiopulmonary failure [1–5]. In neonates, ECMO is mainly used for treating severe respiratory failure in cases of ineffective conventional respiratory support [6]. According to the report, compared with adults and children, neonates with respiratory diseases have the best prognosis after receiving ECMO, with an average survival rate of 74% [7]. The basic principle of ECMO is to drain blood from the body to the outside through arterial and venous cannulation. After artificial membrane oxygenation, oxygenated blood is perfused into the body through the pump to maintain the blood and oxygen supply of various body organs. After providing circulatory and respiratory support for patients with severe cardiopulmonary failure for a long time, the patient’s heart and lungs are fully rested. Neonates with respiratory failure often have severe hypoxemia, acidosis, and pulmonary hypertension, which can lead to cardiac insufficiency and circulatory instability. Therefore, venoarterial (VA) ECMO is generally used in neonates with severe circulatory and respiratory failure [8–10].

The first step of ECMO treatment is to establish adequate circulation access. The right common carotid artery and the right internal jugular vein are generally used for intubating neonates for VA ECMO. During ECMO extubation, the right common carotid artery and the right internal jugular vein are ligated in most centres. However, as the study progressed, concerns about neurological complications of right common carotid artery ligation were raised [11]. With the development of surgical instruments and the improvement of vascular anastomosis techniques, repair of the injured cervical vasculature after ECMO treatment has been attempted in some centres, and the clinical results have been good [11–14]. Because right internal jugular vein anastomosis is more complex, there are few reports of repairing the right internal jugular vein. In our centre, we performed repair operations on the neonates’ right common carotid artery and the right internal jugular vein after ECMO treatment to restore the anatomical integrity and avoid the impact of abnormal blood flow on brain development. This study summarized the early clinical results and surgical experience of repairing the right common carotid artery and the right internal jugular vein after ECMO in neonates at our centre.

## Methods

This study was approved by our hospital ethics committee. Families were told about the process and meaning of the treatment and signed informed consent forms. We retrospectively collected the clinical data of 16 neonates with circulatory and respiratory failure who were treated with ECMO via right common carotid artery and right internal jugular vein cannulation in our hospital from June 2021 to December 2022. There were 11 males and 5 females with an age of 2 days (1–25 days) and a body weight of 3.3 kg (1.7–4.8 kg). The primary disease was neonatal respiratory distress syndrome in 4 patients, severe pneumonia in 2 patients, persistent pulmonary hypertension in 7 patients, and congenital diaphragmatic hernia in 3 patients.

### Method of cannulation of ECMO

All the patients underwent cannulation for ECMO at the bedside in the cardiac intensive care unit. The patient was supine, and then the head was tilted back by padding the shoulders with cotton and turning to the left to expose the right neck fully. A 2 cm incision was made along the transverse line of the neck at 1–2 cm above the right supraclavicular fossa. The platysma and superficial fascia were dissected to expose the leading edge of the sternocleidomastoid muscle. The sternocleidomastoid muscle was bluntly separated to the right to reveal the carotid sheath. The right common carotid artery and the right internal jugular vein were exposed by opening the carotid sheath. Then, the right common carotid artery and the right internal jugular vein were fully dissociated. Two 2 − 0 silk pieces were placed on the central end of the right common carotid artery and the right internal jugular vein, and one piece was placed on a Rumel tourniquet. One 2 − 0 silk piece was placed on the cephalic end of the right common carotid artery and the right internal jugular vein.

**Fig. 1:**
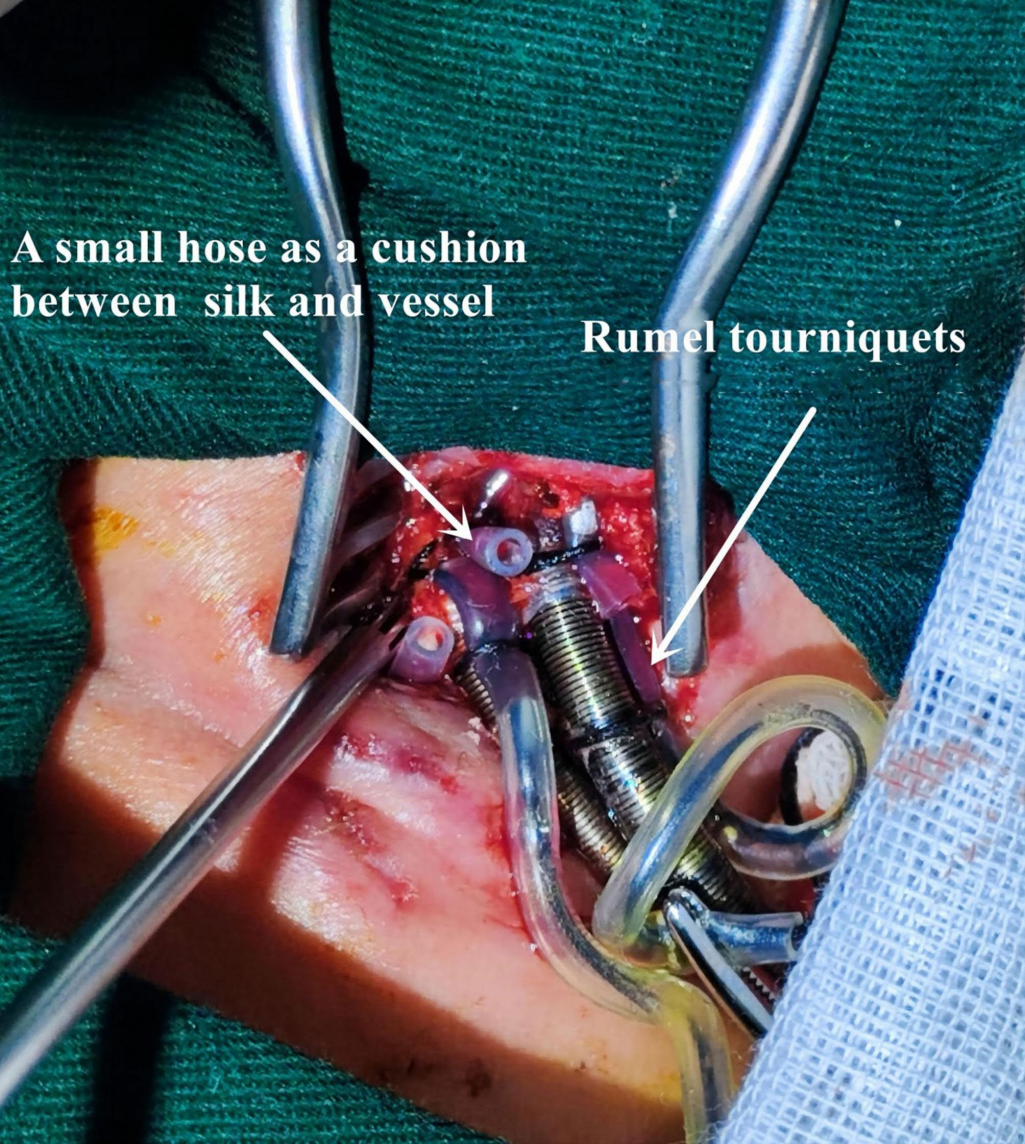
Fixation of the arterial and venous cannulas for ECMO and ligation of the artery and vein of the cephalic end of the heart

The cephalic end of the right common carotid artery was blocked by blocking forceps, and then the central end was blocked. After longitudinally cutting open the artery, an 8 F arterial cannula (Medtronic) was inserted. At the same time, the central end of the occlusion forceps was inserted, and then the arterial cannula was tightened with 2 − 0 silk (Rumel tourniquets). The arterial cannula was fixed with 2 − 0 silk. The cephalic end of the blocking forceps was opened. We used a small hose as a cushion and indwelling 2 − 0 silk thread ligation at the cephalic end of the artery to prevent bleeding and to secure the central end of arterial cannula (Fig. 1). After the arterial cannula was fixed, the core was pulled out, and the ECMO artery and the arterial cannulation were connected. Right internal jugular vein cannulation was performed using the same method and procedure. (10 F vein cannulation, Medtronic)

The ECMO operation was started after ECMO cannulation was established successfully. We confirmed the cannula location via bedside transthoracic echocardiography and chest radiograph examination. After ensuring that the position and flow were satisfactory and that there was no bleeding, we sutured the incision. The artery and vein cannulas were sutured and fixed on the skin.

### Method of ECMO extubation

The anaesthesia process and the position of the patients were the same as the cannulation process. After disinfection, we opened the incision suture to expose the right cervical vessel and cannula. We shut down ECMO and clamped the cannula. Then, we cut the silk of the fixed arteries and veins at the central end of the heart and the silk of the ligating arteries and veins at the cephalic end of the heart.

**Fig. 2:**
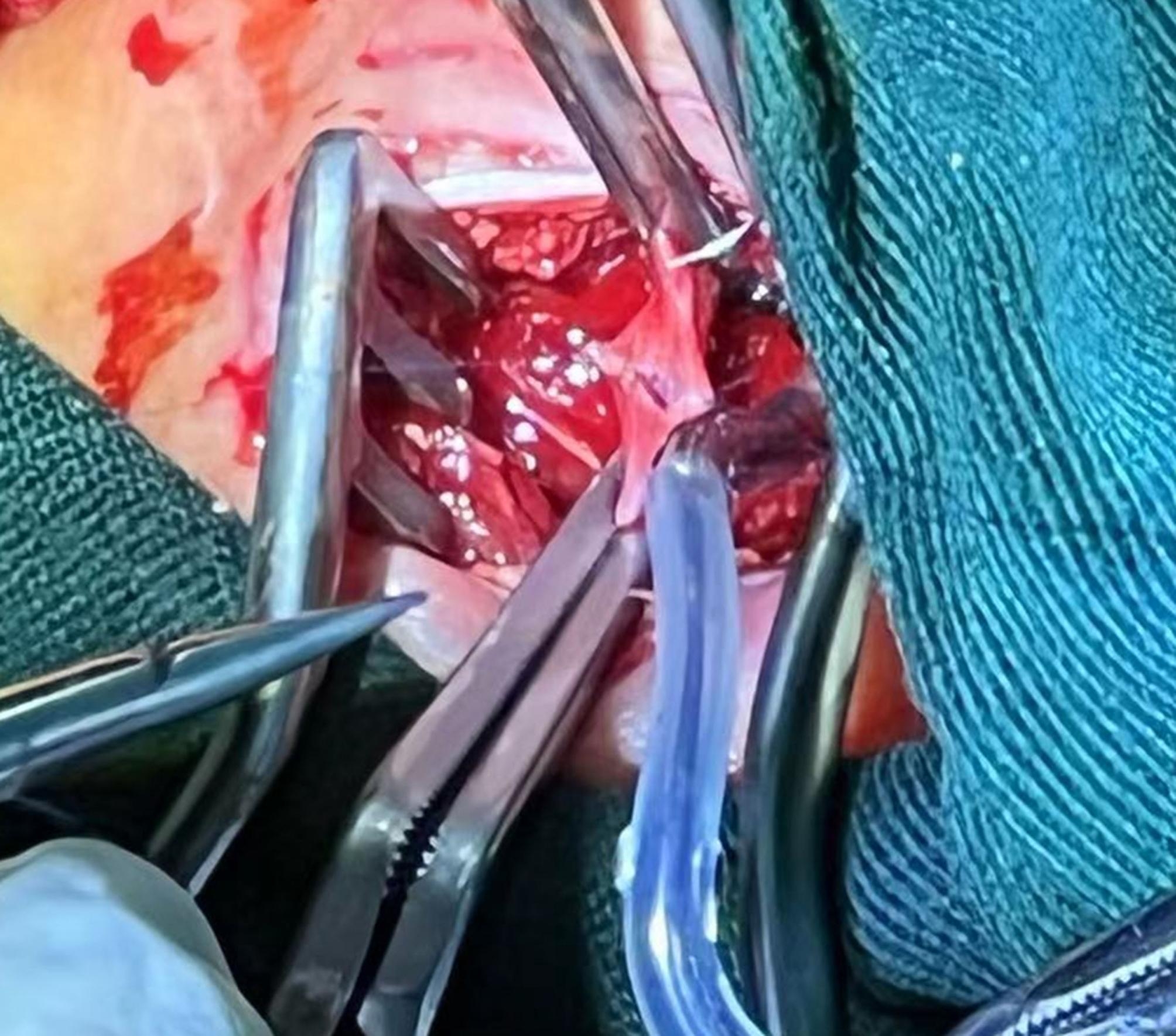
We sutured the vein incision closed with an 8 − 0 Prolene line, and the transverse suture method was used for the anastomosis

**Fig. 3:**
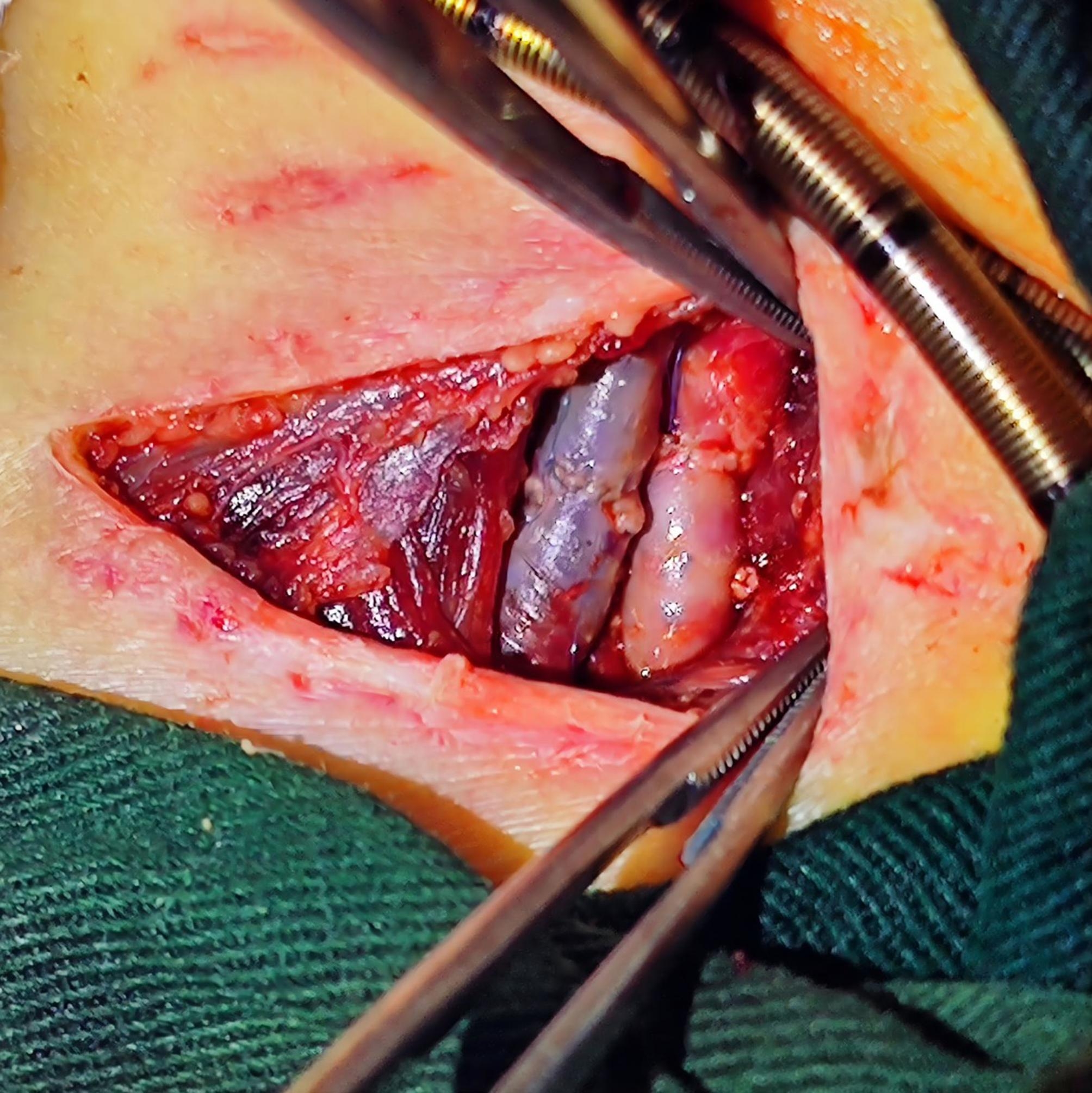
The right common carotid artery and internal jugular vein after repair

Vein cannulation was performed first. After loosening the Rumel tourniquets, we pulled the vein cannula from the right internal jugular vein, tightened the Rumel tourniquets to prevent bleeding, and used the same procedure to pull out the artery cannula. Occlusion forceps were used to clamp the central and cephalic ends of the internal jugular vein, and the Rumel tourniquets were removed. After detecting the patency of the vessels in the central and cephalic ends by using a bougie to dredge, we transversely sutured the vein incision with an 8 − 0 Prolene line. Before tying the knot, the clamp’s central and cephalic ends were opened to vent blood (Fig. 2). Then, we sutured the artery incision by the same method (Fig. 3). The incision was washed with hydrogen peroxide and normal saline successively and then closed with intermittent sutures, and the rubber drainage strip was indwelled. Heparin (1–2 IU/kg/h) was used for anticoagulation, and APTT was maintained for 40–60 s. After recovery of gastrointestinal function, 3–5 mg/kg/d aspirin was used for anticoagulation to prevent thrombosis.

**Fig. 4:**
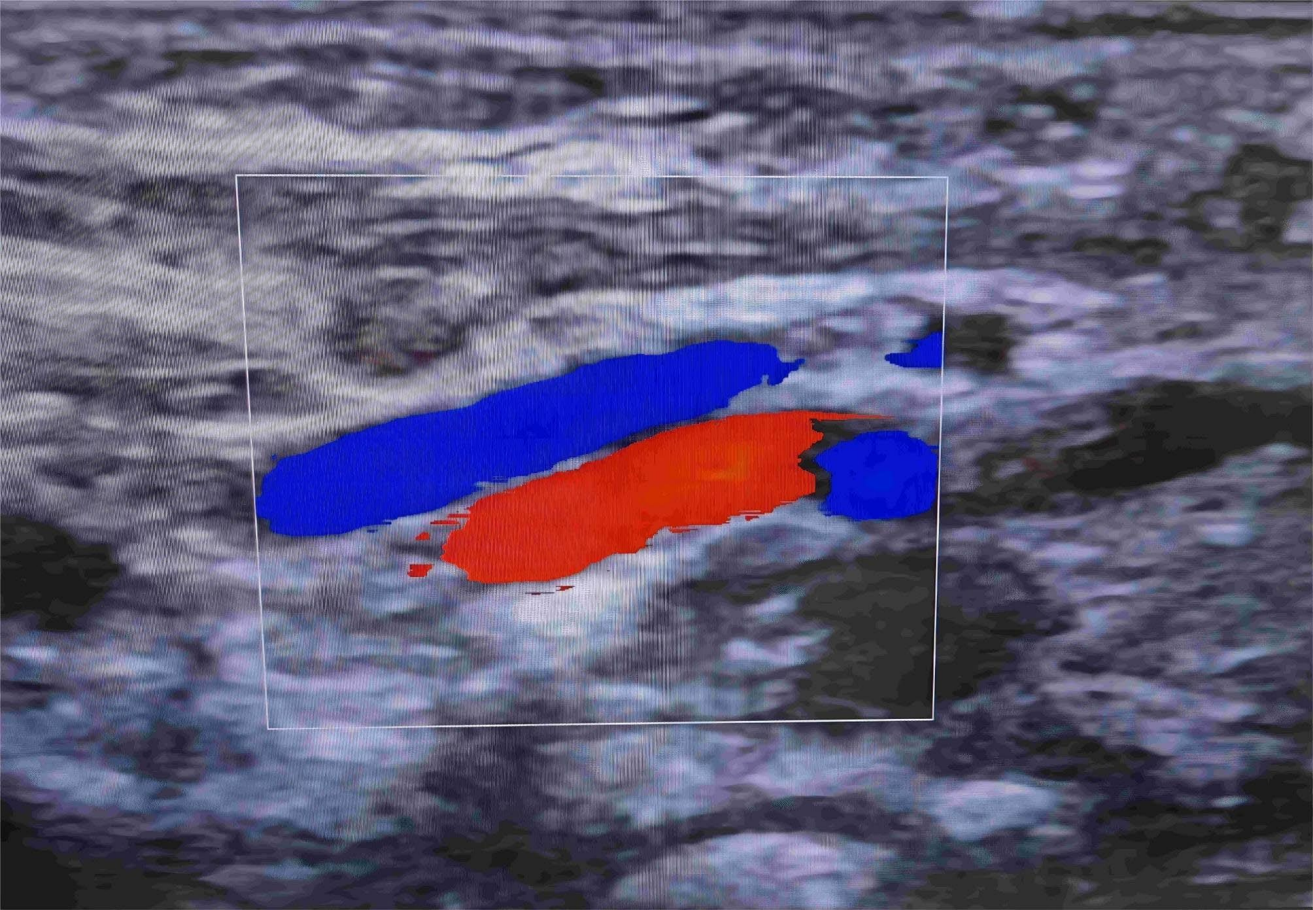
Doppler ultrasound showed that the right common carotid artery and the right internal jugular vein were unobstructed

**Fig. 5:**
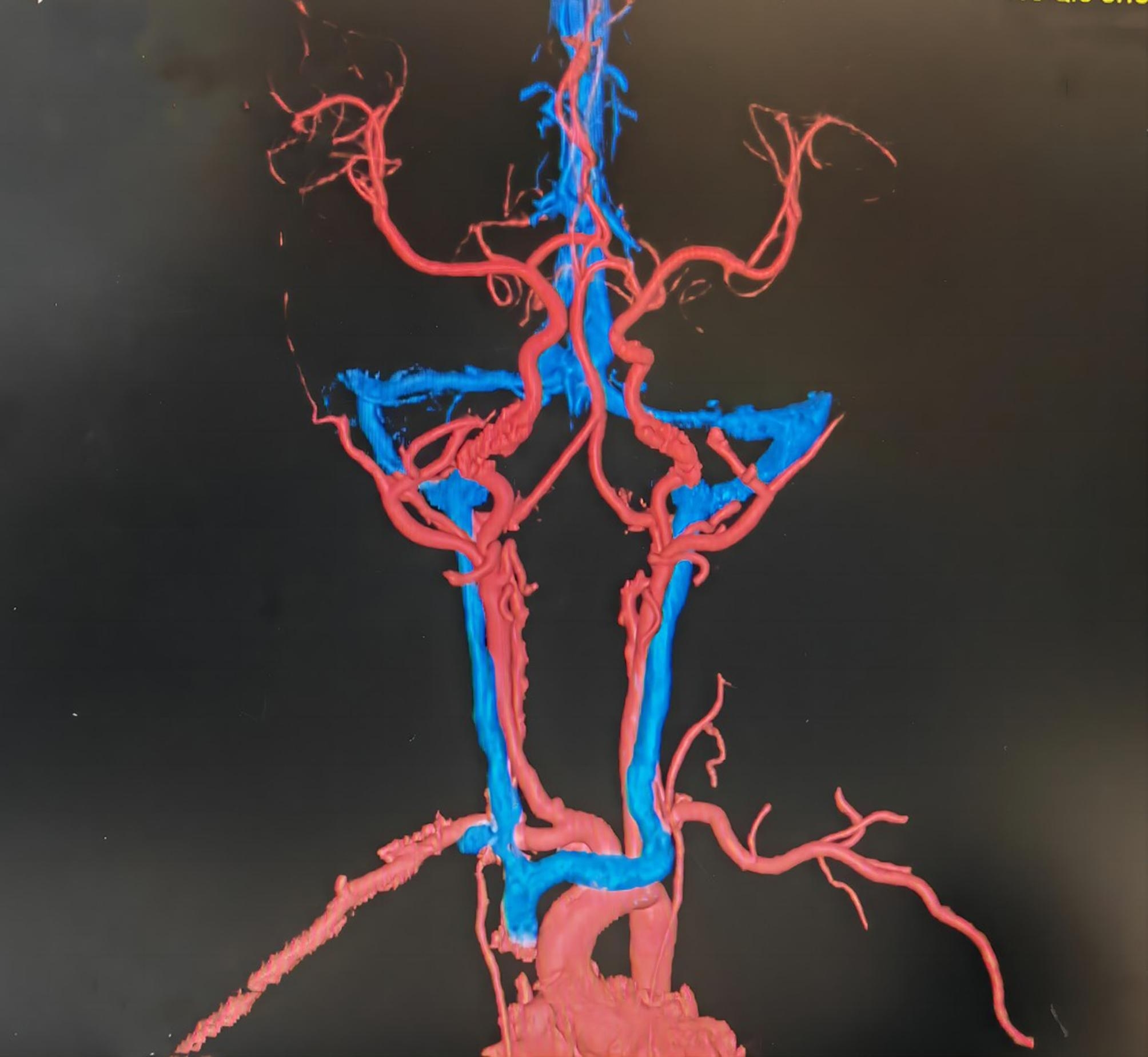
CT showed that the right common carotid artery and the right internal jugular vein were unobstructed

To evaluate the patency of the right cervical vessel in the early period, right cervical vascular ultrasound was performed one week after ECMO withdrawal (Fig. 4). At this time, neck MR angiography was performed for patients who were extubated and had stable respiratory conditions, and neck CT angiography was performed for patients who were not extubated or had unstable respiratory conditions (Fig. 5).

## Results

All patients were successfully intubated with an 8 F artery cannula (Medtronic Company, America) and a 10 F vein cannula (Medtronic Company, America). The depth of the cannula in the right common carotid artery was 2.8–3.5 cm. The depth of the cannula in the right internal jugular vein was 6-7.5 cm. The duration of the ECMO runs was 84 (49, 314) hours. The right common carotid artery and the right internal jugular vein were successfully repaired in 12 patients who were successfully removed from ECMO, and the duration of the ECMO runs was 84 (55, 103) hours. All patients underwent right cervical vascular colour Doppler ultrasound examination at 1 week after the operation. Five patients underwent neck MR angiography examination, and seven patients underwent neck CT angiography examination. The situation of right common carotid artery patency was as follows. Colour Doppler ultrasound showed that the right common carotid artery of 9 patients was unobstructed. For these patients, CT showed mild stenosis in 5 patients, and MR showed mild stenosis in 4 patients. Doppler ultrasound and CT both showed mild stenosis in 1 patient. Doppler ultrasound showed mild stenosis in 1 patient, but MR showed moderate stenosis. Doppler ultrasound and CT both showed an obstruction in 1 patient. The situation of right internal jugular vein patency was as follows. Colour Doppler ultrasound showed that the internal jugular veins of 10 patients were unobstructed. For these patients, CT showed mild stenosis in 6 patients, and MR showed mild stenosis in 4 patients. Doppler ultrasound and MR both showed mild stenosis in 1 patient. Doppler ultrasound showed mild stenosis in 1 patient, but CT showed moderate stenosis. Thrombosis was found in the right common carotid artery of one patient. No thrombosis was found in the right internal jugular vein or intracardiac structure.

## Discussion

Neurological complications after ECMO treatment have always been a concern for doctors [15]. When VA ECMO is used in neonates, there has been controversy over whether the right common carotid artery should be repaired. The blood vessels in the neck of neonates are thin, fragile, and easy to tear. Therefore, repairing the right common carotid artery and the right internal jugular vein after ECMO treatment in neonates is very difficult. Studies have shown that repairing the right common carotid artery and the right jugular vein has the potential risk of embolism, anastomotic burst, and aneurysm [16]. Therefore, the ligation of the right common carotid artery after VA ECMO treatment was normalized in the past, and in most cases, the right common carotid artery is still ligated today.

Many studies have shown that unilateral common carotid artery and internal jugular vein ligation do not increase the risk of neurological complications. Short- and medium-term follow-up studies showed that most patients were in good condition [17–20]. However, some other studies have shown that unilateral common carotid artery and internal jugular vein ligation causes changes in intracranial blood flow, which might affect brain development [11, 21]. DPolito et al. showed that the incidence of neurological complications were higher for VA ECMO than for venovenous (VV) ECMO, which was considered to be caused by ligation of the common carotid artery [22]. In the past ten years, microvascular anastomosis techniques have dramatically improved. Experience with various cardiovascular procedures in infants, such as arterial switches and repair of coarctation of the aortic arch, has shown that most of these vascular anastomoses grow well with the infant. Many surgeons have begun to repair the common carotid artery and achieved high patency rates. Duggan et al. repaired the common carotid artery of 140 neonates treated with VA ECMO, and the images showed that 84% of carotids remained fixed and patent [11]. Spector et al. repaired the common carotid artery of 18 neonates after VA ECMO treatment [12]. After surgery, the blood flow of the common carotid artery of 14 neonates was unobstructed, and the blood flow of the bilateral anterior and middle cerebral arteries was unobstructed. A study by Desai et al. showed that common carotid artery reconstruction was successful in 26 (76%) of 34 children treated with VA ECMO [13]. Sarioglu et al. performed common carotid artery reconstruction in 32 VA ECMO newborns, and the early patency rate was 93% [14].

At present, ligation of the right internal jugular vein after ECMO treatment is the most common strategy in most centres. The reasons for consideration are as follows. (1) Ligation of the right internal jugular vein has little effect on cerebral blood flow due to its compensatory mechanism. (2) Neonatal veins are thinner and more fragile than adult arteries and are more challenging to anastomose. (3) Venous anastomosis is prone to thrombosis, which increases the risk of embolic complications. However, studies have shown that changes in intracranial haemodynamics are closely related to the pathogenesis of a brain injury. Cowan and Thoresen found a marked difference in venous return in neonates after transient obstruction of one or both jugular veins using a continuous wave of a Doppler flowmeter [23]. George et al. found that brain injuries were associated with abnormal venous drainage resulting from ligation of the internal jugular vein [24]. To eliminate the influence of brain injuries or poor brain development caused by ligation of the right common carotid artery and the right internal jugular after ECMO treatment in neonates, the right common carotid artery and the right internal jugular vein were repaired in all neonates who were successfully removed ECMO in our centre. We achieved good early results. The results showed unobstructed arterial blood flow in 9 patients, mild stenosis in 1 patient, moderate stenosis in 1 patient and an obstruction in 1 patient. There was unobstructed venous blood flow in 10 patients, mild stenosis in 1 patient, and moderate stenosis in 1 patient. No thrombosis was found in the right internal jugular vein. Thrombosis was found in the right common carotid artery of one patient. We found that the common carotid artery of this patient was small during ECMO cannulation. The small size of the artery resulted in arterial obstruction after repair.

In the neonate, the common carotid artery and internal jugular vein are slender, with thin vascular walls that are challenging to anastomose, and there is still a high risk of stenosis or occlusion after reanastomosis. To improve the success rate and patency rate of the right common carotid artery and the right internal jugular vein anastomosis, the experience in our centre was as follows. (1) After successful cannulation, a hose pad was placed between the silk and the vessel during fixation of the cannula and ligation of the cephalic end of the artery and vein so that it was not only easier but also did not damage the vessel during the removal of the ligating silk. (2) The surgeon who performed the vascular anastomosis must be skilled in vascular anastomosis. The surgeon who performed vascular anastomosis in our centre had more than ten years of experience in performing paediatric cardiac surgery and was skilled in vascular anastomosis. (3) Before vascular anastomosis, the arterial and venous vascular incisions were thoroughly washed with normal saline to avoid the residue of blood clots and tissue fragments. (4) We used 8 − 0 Prolene line to suture the vascular anastomosis, which could reduce the occurrence of scar formation of the anastomosis as much as possible. It was conducive to long-term vascular growth. (5) Transverse suturing was used during the anastomosis. Compared with longitudinal sutures, transverse sutures could avoid vessel constriction after suturing. During the anastomosis, each stitch should be sure sutured to the intima of the blood vessel to avoid the formation of a haemangioma. (6) After repairing the vasculature, anticoagulant therapy should be performed to avoid thrombosis.

Our study had some limitations. First, this study was a retrospective study, not a prospective study. Second, this study was a single-centre study with a small sample size. Third, the study needed long-term follow-up data.

## Conclusion

Repairing the right common carotid artery and the right internal jugular vein after ECMO in neonates was feasible. Careful surgical anastomosis techniques and standardized postoperative anticoagulation management can ensure early patency. However, long-term follow-up is still needed to confirm their patency.

## Data Availability

The data that support the findings of this study are available on request from the corresponding author. The data are not publicly available due to privacy or ethical restrictions.
